# Risk of suicide attempt and suicide in young adult refugees compared to their Swedish-born peers: a register-based cohort study

**DOI:** 10.1192/j.eurpsy.2022.825

**Published:** 2022-09-01

**Authors:** G. Geirsdottir, E. Mittendorfer-Rutz, R. Amin

**Affiliations:** Karolinska Institutet, Clinical Neuroscience, solna, Sweden

**Keywords:** suicide attempt, Refugee, Unaccompanied minor, Suicide

## Abstract

**Introduction:**

Refugees, especially minors, who often have experienced traumatic events, are a vulnerable group regarding poor mental health. Little is known, however, of their risk of suicidal behaviour as young adults.

**Objectives:**

We aimed to investigate the risk of suicidal behaviour for young adult refugees who migrated as minors. The moderating role of education and history of mental disorders in this association was also investigated.

**Methods:**

In this register linkage study, all 19-30-year-old Swedish-born (n = 1,149,855) and refugees (n = 51,098) residing in Sweden on December 31st, 2009 were included. The follow-up period covered 2010-2016. Cox models were used to calculate hazard ratios (HRs) with 95% confidence intervals (CIs). The multivariate models were adjusted for socio-demographic, labour market marginalisation and health-related factors.

**Results:**

Compared to Swedish-born, the risk of suicide attempt was lower for all refugees (HR 0.78, 95% CI 0.70-0.87), and accompanied refugee minors (HR 0.77, 95% CI 0.69-0.87), but estimates did not differ for unaccompanied refugee minors (HR 0.83, 95% CI 0.62-1.10). Low education and previous mental disorders increased the risk of suicide attempt in both refugees and Swedish-born, with lower excess risks in refugees. Findings for suicide were similar to those of suicide attempt.

**Conclusions:**

Young adult refugees have a lower risk of suicidal behaviour than their Swedish-born peers, even if they have low educational level or have mental disorders. Young refugees who entered Sweden unaccompanied do not seem to be equally protected and need specific attention.

**Disclosure:**

No significant relationships.

